# Role of pharmacogenomics for prevention of hypersensitivity reactions induced by aromatic antiseizure medications

**DOI:** 10.3389/fphar.2025.1640401

**Published:** 2025-08-12

**Authors:** Angel T. Alvarado, Amparo Iris Zavaleta, César Li-Amenero, María R. Bendezú, Jorge A. Garcia, Haydee Chávez, Juan J. Palomino-Jhong, Felipe Surco-Laos, Doris Laos-Anchante, Elizabeth J. Melgar-Merino, Pompeyo A. Cuba-Garcia, Paulina E. Yarasca-Carlos

**Affiliations:** ^1^ Research Unit in Molecular Pharmacology and 4P Medicine, VRI, San Ignacio de Loyola University, Lima, Peru; ^2^ Molecular Biology Laboratory, Faculty of Pharmacy and Biochemistry, National University of San Marcos, Lima, Peru; ^3^ Victor Larco Herrera Hospital, Lima, Peru; ^4^ Faculty of Pharmacy and Biochemistry, San Luis Gonzaga National University of Ica, Ica, Peru; ^5^ Biological Sciences Faculty, San Luis Gonzaga National University of Ica, Ica, Peru

**Keywords:** pharmacogenomics, hypersensitivity reactions, antiseizure medications, epilepsy, clinical implications

## Abstract

Epilepsy is the second most common neurological condition worldwide, characterized by recurrent, unprovoked, self-limiting seizures of genetic, acquired, or unknown origin. The objective was to describe the pharmacogenomic markers associated with hypersensitivity reactions induced by aromatic antiseizure medications. This review explored the pharmacokinetics, pharmacogenomics of *CYP2C9* and *HLA* associated with hypersensitivity reactions, immunopathogenesis and its clinical implications. The included studies applied odds ratio (OR), 95% confidence interval (95% CI) and p value, as association statistics between severe cutaneous adverse reactions (SCARs) including Stevens-Johnson syndrome (SJS) and toxic epidermal necrolysis (TEN). An association study was found between CYP2C19*2 and SCARs induced by carbamazepine, phenytoin and phenobarbital. Five studies of CYP2C9*3 associated with phenytoin-induced SCARs, four studies of CYP2C9*3, HLA-B*13:01, HLA-B*15:02, HLA-B*51:01 and HLA-B*55:01 HLA-B*46:01 and HLA-B*56:02/04 associated with phenytoin-induced SCARs. Likewise, six studies found an association between HLA-B*15:02 and carbamazepine-induced SJS/TEN, four studies associated HLA-A*02:07, HLA-A*24:02, HLA-A*33:03, HLA-B*15:02, HLA-B*44:03 with lamotrigine-induced SCARs, one study associated HLA-A*02:01, HLA-B*35:01, HLA-C*04:01, and HLA-C*08:01 with lamotrigine- and phenytoin-induced SCARs. Three association studies between HLA-A*02:01, HLA-A*11:01, HLA-A*24:02, HLA-B*15:02, HLA-B*38:01, HLA-B*40:02 and HLA-DRB1*03:01 with SCARs induced by carbamazepine, lamotrigine and phenytoin. Published scientific evidence demonstrates that CYP2C9*3 and various *HLA* alleles are associated with severe cutaneous adverse reactions such as toxic epidermal necrolysis and Stevens-Johnson syndrome. Neurologists should consider these allelic variants as predictive and preventive genetic biomarkers of severe adverse reactions to carbamazepine, phenytoin, phenobarbital, and lamotrigine, especially in Asian populations.

## 1 Introduction

The genes *CYP2C9*, *CYP2C19* and *CYP3A4* are associated with variability in plasma levels, pharmacotherapy failure, and adverse drug reactions (ADRs) induced by antiseizure medications (ASMs) (Hirota et al., 2013; Alvarado et al., 2023a). The *CYP3A4* gene has a wild-type CYP3A4*1A allele that configures the CYP3A4*1A/*1A genotype, and this determines the normal metabolic phenotype (Apellániz-Ruiz et al., 2015; Alvarado et al., 2025). Meanwhile, the CYP3A4*20 and CYP3A4*22 alleles constitute the CYP3A4*20/*20 and CYP3A4*22/*22 genotypes, respectively, predicting poor metabolic phenotypes (Apellániz-Ruiz et al., 2015; Zhou et al., 2017). The other gene is *CYP2C9*, which presents the wild-type CYP2C9*1 allele, which constitutes the CYP2C9*1/*1 genotype that predicts the normal metabolic phenotype; Meanwhile, CYP2C9*2 and CYP2C9*3 constitute the genotypes that predict their respective poor metabolic phenotypes (Céspedes-Garro et al., 2015; Alvarado et al., 2019). *CYP2C19* presents the wild-type CYP2C9*1 allele and constitutes the CYP2C19*1/*1 genotype that predicts the normal metabolic phenotype, additionally, the CYP2C19*2 and CYP2C19*3 alleles constitute the genotypes that predict their respective poor metabolic phenotypes that are related to ADRs and toxicity (Dehbozorgi et al., 2018; Maruf et al., 2019).

Likewise, human leukocyte antigens (HLA) that are part of the human major histocompatibility complex (MHC) genes (Phillips et al., 2018), have various alleles that are present in specific populations. The frequency of the HLA-B*15:02 allele is highest in populations from East Asia (6.9%), followed by Oceania (5.4%), South/Central Asia (4.6%) (Chung et al., 2004; Locharernkul et al., 2008; Wang et al., 2011; Gunathilake et al., 2016; Harris et al., 2016), less than 1% in Japanese and approximately 2.5% in Koreans (Phillips et al., 2018), not observed in Africans, less than 1% in African Americans, Caucasians, Hispanic/South Americans, and Middle Eastern populations (Phillips et al., 2018). The HLA-B*15:02 allele is strongly associated with carbamazepine (CBZ)-induced Stevens-Johnson syndrome (SJS) and toxic epidermal necrolysis (TEN) (Chung et al., 2004; Chang et al., 2011; Tangamornsuksan et al., 2013; Phillips et al., 2018; Sung et al., 2020), therefore, the United States Food and Drug Administration (FDA) in 2007 recommended performing a pharmacogenomic test before starting drug treatment (Ferrell and McLeod, 2008). Meanwhile, HLA-A*31:01 is found in Hispanic/South American (6%), Caucasian (3%), Japanese (8%), South Korean (5%) populations, as well as in South and Central Asians (2%) (Phillips et al., 2018). Additionally, the HLA-A*31:01 allele and HLA-A*24:02 have been reported in the Spanish Caucasian population and in other populations (Ramírez et al., 2017). This HLA-A*31:01 is associated with an increased risk of drug reaction with eosinophilia and systemic symptoms (DRESS), and CBZ-induced SJS/TEN (Phillips et al., 2018).

Regarding the adverse drug reaction (ADR), this is a harmful and unintentional response that occurs at standard doses during the treatment, prophylaxis or diagnosis of a disease (Montané and Santesmases, 2020). Cutaneous adverse drug reactions are estimated to be more than 8% of the world’s population susceptible to experiencing them (Błaszczyk et al., 2015), and more than 10% of hospitalized patients suffer from it, but in most cases, these reactions are mild or resolve on their own (Fernández and Pedraz, 2007). ASM-induced cutaneous eruptions occur in 3% (Błaszczyk et al., 2015). These ADRs can be classified into six types, type A, type B, type C reactions or chronic reactions that are associated with side effects to the accumulated dose of the drug over time; Type D is more specific due to the appearance of teratogenesis and carcinogenesis problems; Type E, which occur after the drug is discontinued; Type F, associated with drug interactions that lead to therapeutic failure (Doña et al., 2014; Cardona et al., 2021; Brockow et al., 2023).

Type A reaction represents more than 80% of the total ADR, it is predictable, dose-dependent and, therefore, when administering high doses of drugs, intoxications are observed, at standard doses it manifests as adverse reactions, for example, hair loss due to cytostatics, sedation due to first generation antihistamines. The probability of developing this type of reaction increases with combination therapy, patients with kidney dysfunction, and older adults. Therefore, they are reversible by reducing the dose or discontinuing drug treatment (Pirmohamed et al., 1998; Montané and Santesmases, 2020). On the other hand, type B reaction or hypersensitivity reaction is dose-independent, unpredictable to a drug (Doña et al., 2014; Cardona et al., 2021; Brockow et al., 2023), occur in genetically predisposed subjects (Fernández and Pedraz, 2007; Brockow et al., 2023), and are clinically subdivided into two types: the immediate reaction that occurs in less than 1 h and takes the form of urticaria and anaphylaxis, the delayed reaction occurs after 1 hour and may manifest as severe cutaneous adverse drug reactions (SCARs) (Böhm et al., 2018; Nguyen et al., 2019; Del Pozzo-Magaña and Liy-Wong, 2024). In clinical practice, three main phenotypes of SCARs induced by ASMs are considered Stevens-Johnson syndrome and toxic epidermal necrolysis (SJS/TEN), drug reaction with eosinophilia and systemic symptoms (DRESS)/drug-induced hypersensitivity syndrome (DIHS), and acute generalized exanthematous pustulosis (AGEP) (Böhm et al., 2018; Nguyen et al., 2019; Tempark et al., 2022; Gibson et al., 2023; Del Pozzo-Magaña and Liy-Wong, 2024). SJS and TEN are characterized by sloughing of the epidermis, mucous membranes, and ocular surface through immune mechanisms leading to cell death and necrosis (Pavlos et al., 2012). Among the ASMs that are higher probability to induce hypersensitivity are 15% phenobarbital (PB), 13% phenytoin (PHT), 11% carbamazepine (CBZ) and less than 5% oxcarbazepine (OXC) (Anderson, 2002; Zaccara et al., 2007; Shorvon, 2011; Błaszczyk et al., 2013; Błaszczyk et al., 2015; Mani et al., 2019; Garg et al., 2023; Bataille et al., 2024).

The immunopathogenesis of these reactions is complex. It has been proposed that certain *HLA* alleles interact with drug metabolites or with the unaltered drug, forming complexes that are presented to cytotoxic T lymphocytes (CD8^+^) that release Fas ligand (FasL or CD95L), tumor necrosis factor alpha (TNF-α), interferon gamma (IFN-γ), perforin, granzyme B and granulysin, at the same time, Natural killer (NK) lymphocytes initiate cell death (Su et al., 2017; Sukasem et al., 2018; Stewart et al., 2024). Cell death involves granzyme B, perforins, and caspases 3/7 that induce keratinocyte apoptosis, necroptosis, and epidermal detachment observed in SJS/TEN (Dodiuk-Gad et al., 2015; Charlton et al., 2020; Stewart et al., 2024). The frequency of severe cutaneous adverse drug reactions (SCARs) is 0.4–1.2 cases per million per year (Verma et al., 2013; Tempark et al., 2022), with an annual incidence (proportion of new cases) of 2–7 per million people (Schwartz et al., 2013; Kloypan et al., 2021), 75.7/100,000 in United Kingdom population (Fowler et al., 2019), being major in East Asians (Tempark et al., 2022). The incidence of SJS and TEN among new users of CBZ, LTG, PB, and PHT is between 0.1% and 0.01% (Fowler et al., 2019). The total number of cases or prevalence of SCAR was 0.32/1,000 hospitalizations in Beijing (Li and Ma, 2006), and 50.000 people a year from the use of aromatic antiepileptic drugs in the United Kingdom population (Fowler et al., 2019). The prevalence of SCAR (SJS and TEN) is 67% of 755 cases of severe adverse reactions in Koreans (Kang et al., 2019). The mortality rate for SJS is 1%–5%, and for TEN it is 25%–30% despite the low incidence (Ahmed et al., 2021).

For these reasons, a descriptive review of the current state of knowledge on genes associated with severe cutaneous adverse drug reactions to medications. It is hypothesized that polymorphisms of the *CYP2C9* and *CYP2C19* genes are risk factors associated with hypersensitivity reactions induced by antiseizure medications and has important implications for clinical practice, since it helps predict and prevent severe cutaneous adverse reactions to medications, while personalizing pharmacological treatment guided by the patient’s genotype.

## 2 Pharmacokinetics of aromatic antiseizure medications

This section describes the pharmacokinetics of the main antiepileptic drugs associated with type B hypersensitivity reactions, with particular emphasis on their metabolism. In this regard, carbamazepine (5-H-dibenzazepine-5-carboxamide) is one of the primary drugs related to the induction of hypersensitivity. It is characterized by a chemical structure of the iminostilbene type, derived from tricyclic antidepressants (Chbili et al., 2017), and belongs to class 2 (low solubility, high permeability) according to the Biopharmaceutics Classification System (BCS) (Alvarado et al., 2021). This drug presents two pKa values: pKa_1_ of 2.3 due to the nitrogen of the dibenzazepine ring, and pKa_2_ of 13.9 from the free carboxamide NH_2_ group, predominantly existing in a non-ionized form in the intestinal mucosa, thus favoring its absorption, though with significant intraindividual variability (Alvarado AT. et al., 2022; Alvarado et al., 2023a). To control epilepsy symptoms, the drug must exceed a minimum effective concentration of 4 mg/L but remain below a minimum toxic concentration of 12 mg/L, achieving steady-state plasma concentration (C_ss_) between 21 and 28 days. Its maximum plasma concentration time (t_max_) is 4–8 h (Chbili et al., 2017; Brown et al., 2021). After absorption, it reaches a bioavailability of 70%–80%, circulating bound to albumin and α_1_-acid glycoprotein by 65%–85%. Its volume of distribution (Vd) ranges from 1.4–1.9 L/kg, indicating high lipophilicity, and its free fraction diffuses into the central nervous system and crosses the placental barrier (Alvarado et al., 2023a). In the liver, CBZ undergoes oxidation through three phase I metabolic pathways. The primary pathway involves isoenzymes CYP3A4, CYP2C19, and CYP2C9, converting CBZ to 10,11-epoxycarbamazepine. This metabolite undergoes two subsequent processes: in phase II metabolism, UDP-glucuronosyltransferases UGT2B7 and UGT1A6 transfer a glucuronic acid group from UDP-α-D-glucuronic acid (UDPGA) to the 10,11-epoxycarbamazepine metabolite to form N-β-glucuronide-10,11-epoxycarbamazepine, which is excreted in urine; alternatively, the enzyme epoxide hydrolase converts it to 10,11-dihydro-10,11-trans-dihydroxycarbamazepine (diOH-CBZ). Subsequently, UGT2B7, UGT1A6, and UBG2B transfer a glucuronic acid group to diOH-CBZ, forming O-β-glucuronide of carbamazepine. The second phase I pathway involves CYP3A4 oxidizing CBZ into 2,3-epoxycarbamazepine. The third metabolic pathway converts CBZ to 3-hydroxycarbamazepine *via* CYP3A4, CYP3A7, and CYP2B6 (Skadrić and Stojković, 2020; Brown et al., 2021; Alvarado et al., 2023a). The half-life (t_1/2_) of CBZ is 12–64 h in neonates, 1.9 h in children, and 25–65 h in adults (Alvarado AT. et al., 2022; Alvarado et al., 2023a).

Meanwhile, oxcarbazepine (10,11-dihydro-10-oxo-5H-dibenz [b,f]azepine-5-carboxamide) is a dibenzoazepine derivative, which belongs to class 2 according to the BCS (Shaw and Hartman, 2010) it has a pKa of 13.73, with the non-ionized form that is absorbed in the intestinal mucosa predominating (Antunes et al., 2017; Yang et al., 2023), and does not interact with food nutrients (Flesch, 2004), resulting in a bioavailability of 95% (May et al., 2003). To control the symptoms of epilepsy, the drug must exceed the minimum effective concentration of 5 mg/L and be below the minimum toxic concentration of 30 mg/L to minimize adverse drug reactions (May et al., 2003). The steady-state plasma concentration of the active metabolite 10,11-dihydro-10-hydroxy-carbazepine (MHD) is reached within 2–3 days in patients receiving oxcarbazepine twice daily, and its peak plasma time (t_max_) is 1–3 h (May et al., 2003; Flesch, 2004; Antunes et al., 2017; Yang et al., 2023), AUC is 63.9 μmol·h/L for R-(−)-MHD and 241.0 μmol·h/L for S-(+)-MHD (Flesch et al., 2011; Antunes et al., 2017). Oxcarbazepine and MHD circulate bound to albumin in 59% and 40%, respectively, but do not bind to α_1_-acid glycoprotein, the Vd is 7.8–12.5 L/kg indicating that it crosses biological barriers concentrating in cerebrospinal fluid and placenta (May et al., 2003; Antunes et al., 2017). In phase I metabolism, oxcarbazepine is converted by cytosolic aryl ketone reductase into (S)-(+)-MHD or (R)-(−)-MHD, with approximately 4% subsequently biotransformed to the inactive 10,11-dihydro-10,11-trans-dihydroxycarbamazepine (DHD). In phase II metabolism, UGT2B7 transfers a glucuronic acid group from UDPGA to the MHD metabolite to generate O-β-glucuronide-MHD (May et al., 2003; Flesch, 2004; Yang et al., 2023). The half-life (t_1/2_) of oxcarbazepine is 1–5 h, and of the active metabolite is 7–20 h, but in children it is shorter and in elderly volunteers it is longer (May et al., 2003; Flesch, 2004). Less than 1% of the drug is excreted unchanged, 27% as MHD, and 49% as MHD glucuronides (May et al., 2003). Oxcarbazepine and the active metabolite exhibit linear pharmacokinetics and do not undergo autoinduction (May et al., 2003). *In vitro*, MHD is a weak inducer of UGT and is therefore unlikely to interact with valproic acid and lamotrigine, which are metabolized by the UGT enzyme. Carbamazepine, phenytoin, and phenobarbital have also been shown to reduce plasma MHD levels by 30%–40% when coadministered with oxcarbazepine (Flesch, 2004).

Phenytoin is a hydantoin derivative (5,5-diphenylhydantoin, 5,5′-diphenylimidazolidine-2,4-dione) that belongs to class 2 according to BSC (Guk et al., 2019; Alvarado et al., 2020; Patocka et al., 2020; Alvarado A. et al., 2022). The secondary amino group (R_2_NH) of the hydantoin aromatic ring generates the pKa of 8.3 that allows its absorption in the intestinal mucosa in its non-ionized form, generating a bioavailability of 80% (Milosheska et al., 2015; Alvarado et al., 2020). To control epilepsy symptoms, phenytoin must exceed a minimum effective concentration of 10 mg/L, remain below a minimum toxic concentration of 20 mg/L, and reach a steady-state plasma concentration within 50 days, maintaining levels within the therapeutic range. Its maximum plasma time (t_max_) is 3–8 h (Thaker et al., 2017; Patocka et al., 2020; Alvarado et al., 2023a). After its absorption, it circulates 90% bound to plasma proteins, mainly to albumin, its volume of distribution (Vd) is 0.6–0.8 L/kg indicating that it easily crosses the blood-brain barrier and is distributed in various tissues (Balestrini and Sisodiya, 2018; Patocka et al., 2020; Alvarado et al., 2023a). In phase I metabolism, CYP2C9 and CYP2C19 isoenzymes convert it to 3′,4′-epoxide phenytoin, which then undergoes two processes: conversion to 3′,4′-dihydrodiol phenytoin by epoxide hydrolase; and transformation to 5-(p-hydroxyphenyl)-5-phenylhydantoin (p-HPPH) by CYP2C9 and CYP2C19. This p-HPPH is further biotransformed: by phase I (CYP2C19 and CYP2C9) to 3′,4′-dihydrodiol phenytoin, and by phase II, where UGT1A transfers a glucuronic acid group from UDPGA to p-HPPH to form O-β-glucuronide-phenytoin. The half-life (t_1/2_) is 22 h but can vary from 8–60 h (Lopez-Garcia et al., 2014; Balestrini and Sisodiya, 2018; Alvarado et al., 2020; Alvarado A. et al., 2022). Between 1% and 5% of the drug is excreted unchanged in the urine. At levels below the minimum effective plasma concentration, it is eliminated by first-order kinetics; at higher concentrations, the enzyme system becomes saturated, and it is eliminated by zero-order kinetics (Craig, 2005). Enzyme inhibitor drugs (valproic acid, amiodarone, cimetidine, chloramphenicol, desulfuran, fluconazole, metronidazole, 5-fluorouracil and sulfonamides) generate increased plasma levels of phenytoin that can induce ADRs; While enzyme inducers (alcohol, carbamazepine, phenobarbital, rifampicin, theophylline, and others) generate greater metabolic capacity, decreasing the plasma levels of the drug and which could be the cause of pharmacotherapeutic failure (Craig, 2005).

Lamotrigine is a phenyltriazine derivative [3,5-diamino-6-(2,3-dichlorophenyl)-1,2,4-triazine] and due to its free amino group (primary amino R-NH_2_) of the triazine ring it has a pKa of 5.7 which allows its absorption in the intestinal mucosa in its non-ionized form, it does not interact with food and does not undergo presystemic metabolism (first-pass effect), obtaining an absolute bioavailability of 98% (Garnett, 1997; Mitra-Ghosh et al., 2020; Costa et al., 2024), and its plasma concentrations increase in direct proportion to the administered dose in the range of 50–400 mg (Costa et al., 2024). The minimum effective concentration of lamotrigine is 22 mg/L and the minimum toxic concentration is 34 mg/L, and for clinical efficacy plasma levels must be maintained within the therapeutic range (Yacubian, 2013). The t_max_ is 1–5 h (Garnett, 1997; Costa et al., 2024). After absorption, it circulates bound to plasma proteins (55%), mainly to albumin; Its Vd is 0.9–1.47 L/kg indicating that it crosses the placental barrier, concentrates in the liver, kidneys, breast milk and other tissues (Fillastre et al., 1993; Costa et al., 2024). Transport proteins such as ATP B1 or P-glycoprotein, encoded by the ABCB1 gene (ATP-binding cassette), influence drug distribution, while the human organic cation transporter 1 (hOCT1), encoded by the SLC22A1 gene (solute carriers family 22, member 1), transports lamotrigine to the liver for metabolism (Dickens et al., 2012; Mitra-Ghosh et al., 2020; Zhou et al., 2021). They are metabolized by phase II of glucuronidation, this process is generated when UGT1A4, UGT1A3, and UGT2B7 transfer a glucuronic acid group from UDPGA to lamotrigine, generating either 2-N-glucuronide or 5-N-glucuronide of lamotrigine (Rowland et al., 2006; Milosheska et al., 2016). There is no evidence of autoinduction or saturable metabolism, though plasma levels are altered by enzyme-inducing or -inhibiting drugs (Garnett, 1997; Biton, 2006). The half-life ranges from 24.1 to 35 h (Garnett, 1997). Less than 10% is excreted unchanged, with most eliminated as 2-N-glucuronide of lamotrigine in the urine (Mitra-Ghosh et al., 2020). Valproate inhibits the metabolism of lamotrigine, increasing the plasma level and doubling or tripling its half-life (Faught et al., 1999). While enzyme-inducing drugs (carbamazepine, phenytoin, and primidone) increase metabolism, decreasing plasma levels and half-life of lamotrigine (Fitton and Goa, 1995).

## 3 Pharmacogenomics of aromatic antiseizure medications induced SCARs

### 3.1 *CYP3A4* gene and allelic variants

The *CYP3A4* gene is mapped to the long arm (q) of chromosome 7, region 21.1 (7q21.1), and consists of 13 exons. It contains a 5′ untranslated region (UTR) of 101 nucleotides, a 3′ UTR of 1152 nucleotides, and its spliced mRNA is approximately 2 kb long, encoding the CYP3A4 protein of 503 amino acids with a molecular weight of 57 kDa and a large active site. This enzyme represents 60%–70% of the total CYP450 content in the liver and intestinal enterocytes, respectively, and metabolizes over 50% of clinically used drugs (Rendic, 2002; Plant, 2007; Zhou et al., 2011; Apellániz-Ruiz et al., 2015), and metabolizes approximately between 30% (Fujino et al., 2021) and 60% of clinically used drugs (Klyushova et al., 2022). The wild-type allele CYP3A4*1A predicts a normal metabolic phenotype (Apellániz-Ruiz et al., 2015), while the reduced function alleles are CYP3A4*2 caused by the thymine (T) change thymine (T) to cytosine (C) substitution at position 15722 (15722T>C) in exon 7; CYP3A4*3 (23181T>C); CYP3A4*22 (15389C>T) (Zhou et al., 2017; Zhou et al., 2019); and CYP3A4*20, which results from the insertion of a single adenine base causing a frameshift and a premature stop codon (25898_25899insA) (Apellániz-Ruiz et al., 2015; Alvarado et al., 2023b). Carriers of the CYP3A4*20 and CYP3A4*22 alleles, corresponding to the CYP3A4*20/*20 and CYP3A4*22/*22 genotypes, are classified as poor metabolizers (PM), with absent or reduced drug metabolism, leading to increased serum drug levels beyond the minimum toxic concentration and predisposing them to adverse drug reactions (Alvarado et al., 2023b; Collins and Wang, 2022).

### 3.2 *CYP2C9* gene and allelic variants

The *CYP2C9* gene is mapped to the long arm (q) of chromosome 10, region 24, spanning 500 kb (10q24), and contains 9 exons. The wild-type allele, CYP2C9*1, forms the CYP2C9*1/*1 genotype, with carriers considered normal metabolizers. This gene encodes the CYP2C9 protein, the second most abundant enzyme within the CYP450 family, representing approximately 10% of all CYP450 enzymes in hepatocyte microsomes (Céspedes-Garro et al., 2015; Maruf et al., 2019; Skadrić and Stojković, 2020; Karnes et al., 2021). More than 61 allelic variants have been described, with decreased-function alleles including: CYP2C9*2 (3608C>T), characterized by a cytosine (C) to thymine (T) substitution at position 3608 in exon 3, resulting in an arginine (Arg) to cysteine (Cys) change at position 144 (Arg144Cys) (de Andrés et al., 2021). CYP2C9*3 (42614A>C), caused by an adenine (A) to cytosine (C) transversion at position 42614 in exon 7, leading to an isoleucine (Ile) to leucine (Leu) substitution at codon 359 (Ile359Leu), altering the enzyme’s active site (de Andrés et al., 2021). CYP2C9*4 is expressed by the change of thymine (T) to cytosine (C) at position 1,076 of the nucleotide sequence (1076T>C;) (Claudio-Campos et al., 2017; Alvarado et al., 2019; Maruf et al., 2019; Skadrić and Stojković, 2020); CYP2C9*5 is caused by the change of cytosine (C) to guanine (G) at position 42619 (42619C>G) (Kidd et al., 2001), meanwhile, CYP2C9*6 (10601 del A) is characterized by splicing deletion causing a frame shift resulting in a truncated protein (de Andrés et al., 2021).

### 3.3 *CYP2C19* gene and allelic variants

The *CYP2C19* gene is mapped to the long arm (q) of chromosome 10, region 24.1 (10q24.1), with a sequence of 1473 base pairs comprising 9 exons and 8 introns. The wild-type allele, CYP2C19*1, forms the CYP2C19*1/*1 genotype, predicting a normal metabolic phenotype. It encodes the CYP2C19 protein composed of 490 amino acid residues (Saeed and Mayet, 2013; Maruf et al., 2019; Skadrić and Stojković, 2020). Among the main null or reduced-activity alleles are: CYP2C19*2 (19154G>A), caused by a guanine (G) to adenine (A) transition at position 19154 in exon 5 (de Andrés et al., 2021), creating an aberrant splice site that alters the mRNA reading frame from amino acid 215, producing a premature stop codon after 20 amino acids (Dehbozorgi et al., 2018; Maruf et al., 2019; Skadrić and Stojković, 2020). CYP2C19*3 (17948G>A), featuring a mutation at position 17948 in exon 4 (de Andrés et al., 2021), resulting in a premature stop codon (Lee, 2013; Saeed and Mayet, 2013; Dehbozorgi et al., 2018; Maruf et al., 2019); CYP2C19*4 (80161A>G); CYP2C19*5 (90033C>T), located in the heme-binding region, causing an Arg433Trp substitution (Lee, 2013; de Andrés et al., 2021). CYP2C19*6 (12748G>A) in exon 3, resulting in an Arg132Gln substitution; and CYP2C19*7 (19294T>A), affecting the 5′ donor splice site of intron 5 (Lee, 2013; Alvarado et al., 2023a). Table 1 describes and summarizes the main alleles, genotypes and intermediate or poor metabolic phenotypes.

**TABLE 1:** Alleles, genotypes, normal, intermediate and poor metabolic phenotypes.

Alleles	Rs ID	Nucleotide change (cDNA)	Allele functional status	Genotype	Phenotype	References
Gene/location: *CYP3A4*/7q21.1						
CYP3A4*1A		None	Normal	CYP3A4*1A/*1A	NM	Apellániz-Ruiz et al. (2015)
CYP3A4*2	rs55785340	15722T>C	Decreased	CYP3A4*2/*2	IM	Zhou et al., 2017; Zhou et al., 2019
CYP3A4*3	rs4986910	23181T>C	Decreased	CYP3A4*3/*3	PM	
CYP3A4*20	rs67666821	25898_25899insA	Decreased	CYP3A4*20/*20	PM	Apellániz-Ruiz et al. (2015)
CYP3A4*22	rs35599367	15389C>T	Decreased	CYP3A4*22/*22	PM	Zhou et al., 2017; Zhou et al., 2019
Gene/location: *CYP2C9*/10q24						
CYP2C9*1		None	Normal	CYP2C9*1/*1	NM	Skadrić and Stojković (2020)
CYP2C9*2	rs1799853	3608C>T	Decreased	CYP2C9*1/*2	IM	de Andrés et al. (2021)
				CYP2C9*2/*2	IM	
CYP2C9*3	rs1057910	42614A>C	Decreased	CYP2C9*1/*3	IM	de Andrés et al. (2021)
				CYP2C9*2/*3	PM	
				CYP2C9*3/*3	PM	
CYP2C9*5	rs28371686	42619C>G	Decreased	CYP2C9*5/*5	PM	Karnes et al. (2021)
CYP2C9*6	rs9332131	10601 delA	Decreased	CYP2C9*6/*6	PM	
Gene/location: *CYP2C19*/10q24.1						
CYP2C19*1	rs3758581	80161A>G	Normal	CYP2C19*1/*1	NM	Maruf et al. (2019)
CYP2C19*2	rs4244285	19154G>A	Decreased	CYP2C19*1/*2	IM	de Andrés et al. (2021)
				CYP2C19*2/*2	PM	
CYP2C19*3	rs4986893	17948G>A	Decreased	CYP2C19*2/*3	PM	de Andrés et al. (2021)
				CYP2C9*3/*3	PM	
CYP2C19*4	rs3758581	80161A>G	Decreased	CYP2C19*4/*4	PM	Lee (2013)
CYP2C19*5	rs56337013	90033C>T	Decreased	CYP2C19*5/*5	PM	Lee (2013)
CYP2C19*6	rs72552267	12748G>A	Decreased	CYP2C19*6/*6	PM	Lee (2013)
CYP2C19*7	rs72558186	19294T>A	Decreased	CYP2C19*7/*7	PM	Lee (2013)

Abbreviations: MN, normal metabolizer; IM, intermediate metabolizer; PM, poor metabolizer.

**FIGURE 1:**
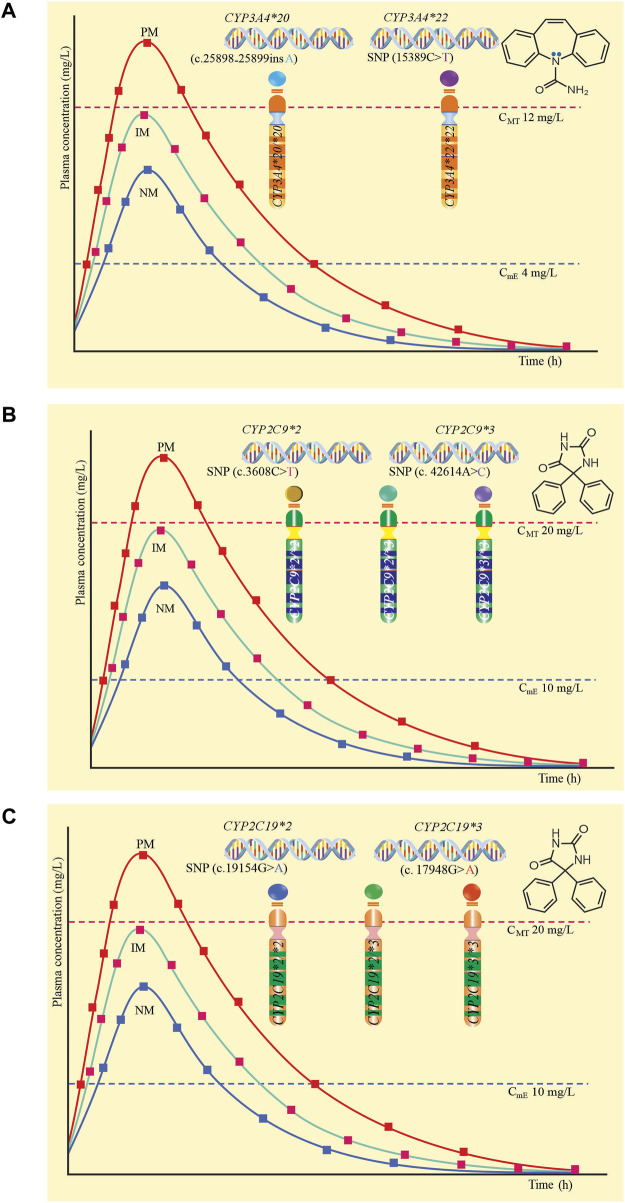
Plasma levels according to genotype and poor metabolic phenotype. **(A)** CYP3A4*20 and CYP3A4*22 and their genotypes CYP3A4*20/*20 and CYP3A4*22/*22. **(B)** CYP2C9*2 and CYP2C9*3 and their genotypes CYP2C9*2/*2, CYP2C9*2/*3 and CYP2C9*3/*3. **(C)** CYP2C19*2 and CYP2C19*3 and their genotypes CYP2C19*2/*2, CYP2C19*2/*3 and CYP2C19*3/*3.

Figure 1A proposes CYP3A4*20 and CYP3A4*22 alleles that configure the CYP3A4*20/*20 and CYP3A4*22/*22 genotypes, which predict poor metabolizers (PM), therefore, in this group of patients, metabolism is null, increasing serum levels of the drug beyond the minimum toxic concentration (12 mg/L), predisposing to adverse drug reactions induced by carbamazepine (Alvarado et al., 2023b; Collins and Wang, 2022). Figure 1B proposes the CYP2C9*2 and CYP2C9*3 alleles and their respective CYP2C9*2/*2, CYP2C9*2/*3, and CYP2C9*3/*3 genotypes, which predict PM, and in which there is a risk of phenytoin-induced adverse drug reactions (Maruf et al., 2019; Skadrić and Stojković, 2020). Figure 1C proposes the CYP2C19*2 and CYP2C19*3 alleles that configure their CYP2C19*2/*2, CYP2C19*2/*3 and CYP2C19*3/*3 genotypes that predict PM, therefore, decreasing the metabolism of the drug, increasing the plasma level and generating adverse reactions (Maruf et al., 2019; Skadrić and Stojković, 2020). The plasma level curves of a normal metabolizer (NM) and an intermediate metabolizer (IM) are also compared.

## 4 Pharmacogenomics of *CYP2C9/CYP2C19* associated with SCAR induced by aromatic antiseizure medications

The frequency of the CYP2C9*2 allele in African Americans represents 3%, and between 3% and 11% in Caucasians, while CYP2C9*3 is present in 1.3% of African Americans and between 3% and 16% in Caucasians (de Andrés et al., 2021). The frequency of CYP2C19*2 in Africans is 17%, African Americans 18%, American population 11%, Central/South East Asia 33%, East Asia 30% and in Europeans 15%, while CYP2C19*3 is only found in Central/South East Asia (1%) and East Asia (7%) (Koopmans et al., 2021).

Prior knowledge of these allele frequencies allows us to predict which ethnic group, admixture, or population is more susceptible to experiencing adverse reactions and severe cutaneous adverse drug reactions (SCARs) induced by aromatic antiseizure medications (ASMs). The frequency in Latin America is variable, where the population is admixture of tricontinental (European, African and Asian) and Amerindian ancestry (Koopmans et al., 2021). Table 2 summarizes and describes the articles (eleven) with the highest quality and scientific evidence, given that they applied association statistics and found a higher risk between allelic variants and severe cutaneous adverse drug reactions.

**TABLE 2:** Allelic variants associated with severe cutaneous adverse drug reactions induced by antiseizure medications of aromatic structures.

Title	Author	Study type	Drug	Allelic variant	SJS/TEN associated with CYP allelic and induced by ASMs	Conclusions and application
Association of galactose single-point test levels and phenytoin metabolic polymorphisms with gingival hyperplasia in patients receiving long-term phenytoin therapy.	Lin et al. (2008)	Prospective analysis.	PHT	*CYP2C9* *CYP2C19*	R-5-(4′-hydroxyphenyl)-5-phenylhydantoin (R-HPPH) is associated with a higher incidence of gingival hyperplasia (OR: 1.0; 95% CI: 1.00–1.03) and PHT (OR: 1.09; 95% CI: 1.00–1.19). R-HPPH (73.92 ± 48.14 ng/mL; p = 0.03) was lower in CYP2C19 poor metabolizers (OR: 0.76; 95% CI: 0.22–2.66; p = 0.67) and CYP2C9 poor metabolizers (OR: 0.44; 95% CI: 0.11–1.78; p = 0.25), but no association was found between genotype and gingival hyperplasia.	Elevated plasma levels of the metabolite phenytoin are associated with a higher incidence of phenytoin-induced gingival hyperplasia.
Phenobarbital-induced severe cutaneous adverse drug reactions are associated with CYP2C19*2 in Thai children.	Manuyakorn et al. (2013)	Cases and controls	CBZ PHT PHB	CYP2C19*2	CYP2C19*2 carriers are more likely to develop CBZ- or PHT-induced SCARs than CYP2C19*1 carriers (OR: 2.5; 95% CI: 0.96–67.3; p = 0.06). CYP2C19*2 carriers are at increased risk for PHB-induced SCARs (OR: 4.5; 95% CI: 1.17–17.37; p < 0.03).	The CYP2C19*2 variant may be a genetic biomarker that predisposes to SCARs due to phenobarbital.
Genetic variants associated with phenytoin-related severe cutaneous adverse reactions.	Chung et al. (2014)	Cases and controls	PHT	CYP2C9*3	CYP2C9*3 showed association with PHT-induced SCARs (OR: 12; 95% CI: 6.6–20; p = 1.1 × 10^−17^).	The CYP2C9*3 allelic variant decreases PHT metabolism and is associated with severe cutaneous adverse reactions.
Association analysis of CYP2C9*3 and phenytoin-induced severe cutaneous adverse reactions (SCARs) in Thai epilepsy children.	Suvichapanich et al. (2015)	Cases and controls	PHT PHB	CYP2C9*3	CYP2C9*3 was associated with PHT-induced SCARs (OR: 14.52; 95% CI: 1.18^−^ ∞ ; p = 0.044). CYP2C9*3 was not associated with PHB-induced SCARs.	CYP2C9*3 is a reasonable predictive biomarker to anticipate phenytoin-induced SCARs.
Influence of genetic and non-genetic factors on phenytoin-induced severe cutaneous adverse drug reactions.	Yampayon et al. (2017)	Cases and controls	PHT	CYP2C9*3	CYP2C9*3 associated with PHT-induced SJS (OR: 10.41; 95% CI: 2.06–55.42; p = 0.0042). HLAB*13:01, HLAB*56:02/04 and CYP2C19*3 (OR: 13.29; p = 0.0001; OR: 56.23; p = 0.0007 and OR: 6.75; p = 0.0414, respectively).	The combination of biomarkers could be potential predictors of PHT-induced SCARs.
Association of CYP2C9*3 with phenytoin-induced Stevens-Johnson syndrome and toxic epidermal necrolysis: A systematic review and meta-analysis	Wu et al. (2018)	A systematic review and meta-analysis	PHT	CYP2C9*3	CYP2C9*3 associated with SJS/TEN when compared with controls (OR: 8.93; 95% CI 2.63–30.36; p = 0.0005) with substantial heterogeneity (I^2^ = 46%) and population controls (OR: 8.88; 95% CI: 5.01–15.74; p < 0.00001).	CYP2C9*3 is a predictive genetic biomarker for PHT-induced SJS/TEN. However, large-scale prospective multicenter observational studies are recommended.
HLA Alleles and CYP2C9*3 as Predictors of Phenytoin Hypersensitivity in East Asians.	Su et al. (2019)	Cases and controls	PHT	CYP2C9*3 HLA-B*13:01 HLA-B*15:02 HLA-B*51:01	An association was detected between CYP2C9*3/HLA-B*13:01, HLA-B*15:02/HLA-B*51:01 and PHT-SCARs (OR: 4.55; 95% CI: 1.44–14.41; p = 0.01).	The combination of HLA and *CYP2C9* risk alleles are potential predictive genetic biomarkers for preventing PHT-induced hypersensitivity in Asians.
Associations of CYP2C9 and CYP2C19 Pharmacogenetic Variation with Phenytoin-Induced Cutaneous Adverse Drug Reactions.	Fohner et al. (2020)	Retrospective cohort	PHT	CYP2C9*3	CYP2C9*3 is associated with PHT-induced SCARs (OR: 4.47; 95% CI: 1.64–11.69; p < 0.01). Asians had 3.70 times higher odds of SCARs than non-Hispanic Caucasians (95% CI: 0.95–12.13; p = 0.04).	The CYP2C9*3 allelic variant may increase the risk of PHT-induced cutaneous adverse events in the absence of the HLA-B*15:02 risk allele.
HLA-B*51:01 and CYP2C9*3 Are Risk Factors for Phenytoin-Induced Eruption in the Japanese Population: Analysis of Data From the Biobank Japan Project.	Hikino et al. (2020)	Cases and controls	PHT	CYP2C9*3 HLA-B*51:01	CYP2C9*3 is associated with PHT-induced rash (OR: 7.05; 95% CI: 2.44–20.4; p = 0.0022). HLA-B*51:01 also showed association (OR: 3.19; 95% CI: 1.37–7.48; p = 0.010).	Before starting treatment with phenytoin, a CYP2C9*3 and HLA-B*51:01 test should be indicated to reduce cutaneous adverse reactions.
Genetic and clinical risk factors associated with phenytoin-induced cutaneous adverse drug reactions in Thai population.	Sukasem et al. (2020)	Cases and controls	PHT	CYP2C9*3 HLA-B*46:01 HLA-B*56:02/04	CYP2C9*3 carriers have an increased risk of PHT-induced SJS/TEN (OR: 4.80; 95% CI: 0.960–23.99; p = 0.056). HLA-B*56:02/04 is associated with PHT-induced DRESS/DHS (OR: 29.312; 95% CI: 1.213–707.994; p = 0.038). HLA-B*46:01 (OR: 2.341; 95% CI: 1.078–5.084; p = 0.032).	The alleles studied contributed to the risk of PHT-induced ADRs. Studies with a larger sample size are proposed to confirm these findings.
Association of HLA-B*51:01, HLA-B*55:01, CYP2C9*3, and Phenytoin-Induced Cutaneous Adverse Drug Reactions in the South Indian Tamil Population.	John et al. (2021)	Cases and controls	PHT	CYP2C9*3 HLA-B*55:01 HLA B*51:01	CYP2C9*3 is associated with SCARs by PHT (OR: 12.00; 95% CI: 2.759–84.87; p = 0.03). CYP2C9*3 and HLA-B*55:01 with SCARs (OR: 12.45; 95% CI: 1.138–136.2; p = 0.003) and maculopapular rash due to PHT (OR: 4.041; 95% CI: 1.125–15.67; p = 0.035). HLA-B*51:01/PHT-SCAR (OR: 6.273; 95% CI: 2.24–16.69; p = <0.001) and HLA B*51:01/PHT-SCARs (OR: 2.323; 95% CI: 1.22–5.899; p = 0.037).	These alleles are risk factors for PHT-induced adverse reactions in the Tamil population of southern India. Further studies with larger sample sizes are recommended to determine the clinical relevance of these alleles.

Abbreviations: ASMs, antiseizure medications; CBZ, carbamazepine; PHT, phenytoin; PHB, phenobarbital; DRESS, drug rash with eosinophilia and systemic symptoms; DHS, drug hypersensitivity syndrome; SCARs, severe cutaneous adverse drug reactions; SJS/TEN, Stevens-Johnson syndrome (SJS) and toxic epidermal necrolysis (TEN); OR, odds ratio; 95% CI, 95% confidence interval.

## 5 The important role of pharmacogenomics diversity related with AEDs-induced SCARs in many populations

The human leukocyte antigen type B*15:02 (HLA-B*15:02) and HLA-A*31:01 alleles are used as pharmacogenomic biomarkers to predict the risk of carbamazepine-induced hypersensitivity reactions (Gunathilake et al., 2016). HLA-B*15:02 is associated with Stevens-Johnson syndrome (SJS) and toxic epidermal necrolysis (TEN) induced by carbamazepine (Hung et al., 2006; Wang et al., 2011), oxcarbazepine and phenytoin (Locharernkul et al., 2008; Kim et al., 2018), and lamotrigine (Zeng et al., 2015). Meanwhile, the HLA-A*31:01 allele is associated with drug reaction with eosinophilia and systemic symptoms (DRESS) syndrome, postoperative myalgic pain syndrome (PMS), and additionally with SJS/TEN (Gunathilake et al., 2016). Table 3 summarizes the *HLA* allelic variants associated with severe cutaneous adverse induced by antiseizure medications.

**TABLE 3:** HLA allelic variants associated with severe cutaneous adverse drug reactions induced by antiseizure medications.

Title	Author	Study type	Drug	HLA allele type	SJS/TEN associated with HLA and induced by ASMs	Conclusions and application
HLA-A 31:01 and HLA-B 15:02 as genetic markers for carbamazepine hypersensitivity in children.	Mehta et al. (2009)	Cases and controls	CBZ	HLA-B*15:02	HLA-B*15:02 associated with CBZ-induced SJS (OR: 71.40; (95% CI: 3.0–16.98; p = 0.0014).	This study suggests that HLA-B*15:02 is associated with carbamazepine-induced SJS in Indian patients.
Frequency of the HLA-B*1,502 allele contributing to carbamazepine-induced hypersensitivity reactions in a cohort of Malaysian epilepsy patients.	Then et al. (2011)	Cases and controls	CBZ	HLA-B*15:02	HLA-B*15:02 associated with CBZ-induced SJS/TEN (p = 0.0006).	Association of HLA-B*15:02 with CBZ-induced SJS/TEN was confirmed in Malay and Chinese epilepsy patients.
Association of HLA-B*1,502 allele with carbamazepine-induced toxic epidermal necrolysis and Stevens-Johnson syndrome in the multi-ethnic Malaysian population.	Chang et al. (2011)	Cases and controls	CBZ	HLA-B*15:02	HLA-B*15:02 associated with SJS/TEN by CBZ in Malaysian patients (75.0%; OR: 16.15; 95% CI: 4.57–62.4; p = 7.87 × 10^−6^) and 15.7% in controls.	In Malaysian patients, they can be used as a genetic marker and help prevent the onset of SJS/TEN.
Strong association between HLA-B*1,502 and carbamazepine-induced Stevens-Johnson syndrome and toxic epidermal necrolysis in mainland Han Chinese patients.	Zhang et al. (2011)	Cases and controls	CBZ	HLA-B*15:02	HLA-B*15:02 associated with CBZ-induced SJS/TEN (p < 0.01) in patients (94.1%).	An association between HLA-B*15:02 and CBZ-induced SJS/TEN was found in the Han population of central and northern China.
HLA-B*1,502 strongly predicts carbamazepine-induced Stevens-Johnson syndrome and toxic epidermal necrolysis in Thai patients with neuropathic pain.	Kulkantrakorn et al. (2012)	Cases and controls	CBZ	HLA-B*15:02	HLA-B*15:02 associated with CBZ-induced SJS/TEN (OR: 75.4; 9`5%CI: 13.0–718.9; p < 0.001).	In Thai patients with neuropathic pain, it can be used as a genetic biomarker for CBZ-induced SJS/TEN.
HLA-A 31:01 and HLA-B 15:02 as genetic markers for carbamazepine hypersensitivity in children.	Amstutz et al. (2013)	Cases and controls	CBZ	HLA-B*15:02	*HLA-B 15:02* was associated with SJS/TEN by CBZ (OR: 38.6; p = 0.002).	In North American pediatric patients of diverse ethnic origins, the HLA-B*15:02 allele is a predictor of CBZ-induced hypersensitivity reactions.
HLA-A*02:01:01/-B*35:01:01/-C*04:01:01 haplotype associated with lamotrigine-induced maculopapular exanthema in Mexican Mestizo patients.	Fricke-Galindo et al. (2014)	Cases and controls	LTG PHT	HLA-C*08:01 HLA-A*02:01 HLA-B*35:01 HLA-C*04:01	The association between HLA-C*08:01 and PHT-induced MPR was higher than in the PHT-tolerant group (pc = 0.0179) or the Mexican mestizo (MM) population (pc < 0.0001). HLA-A*02:01:01/-B*35:01:01/-C*04:01:01 and LTG-induced MPR (pc = 0.0048 for the LTG-tolerant groups and pc < 0.0001 for the MM population).	HLA allelic variants could be considered as biomarkers of LTG- and PHT-induced maculopapular rash. Further research is recommended to confirm these findings.
The HLA-A*2402/Cw*0102 haplotype is associated with lamotrigine-induced maculopapular eruption in the Korean population.	Moon et al. (2015)	Cases and controls	LTG	HLA-A*24:02	A significant association was identified between HLA-A*24:02 and LTG-induced MPR, compared with the LTG-tolerant group (OR: 4.09, p = 0.025) and the general Korean population (OR 3.949, p = 0.005).	The results suggest that HLA-A*24:02 is associated with LTG-induced MPR in the Korean population.
Association of HLA-B*1,502 allele with lamotrigine-induced Stevens-Johnson syndrome and toxic epidermal necrolysis in Han Chinese subjects: a meta-analysis.	Zeng et al. (2015)	Meta-analysis	LTG	HLA-A*24:02	An association was found between HLA-B*15:02 and LTG-induced SJS/TEN (OR = 4.98, 95% CI 1.43–17.28, p < 0.05).	There is an association with the risk of lamotrigine-induced SJS/TEN. Future studies with larger sample sizes are suggested to verify these results.
HLA Allele Frequencies in 5802 Koreans: Varied Allele Types Associated with SJS/TEN According to Culprit Drugs.	Park et al. (2016)	Retrospective	LTG	HLA-B*44:03	An association between HLA-B*44:03 and LTG-induced SJS/TEN was reported (OR = 12.75, 95% CI 1.03–157.14, p = 0.053).	HLA-B*44:03 may be associated with lamotrigine-induced SJS/TEN.
Association of HLA-A and HLA-B Alleles with Lamotrigine-Induced Cutaneous Adverse Drug Reactions in the Thai Population.	Koomdee et al. (2017)	Cases and controls	LTG	HLA-A*02:07 HLA-A*33:03 HLA-B*15:02 HLA-B*44:03	An association was found between HLA-A*02:07 and HLA-B*15:02 SCARs induced by LTG than in tolerant controls (OR = 7.83, 95% CI 1.60–38.25, p = 0.013, and OR = 4.89, 95% CI 1.28–18.67, p = 0.014). HLA-A*33:03, HLA-B*15:02 and HLA-B*44:03 associated with LTG-induced MPR than in tolerant controls (OR = 8.27, 95% CI 1.83–37.41, p = 0.005; OR = 7.33, 95% CI 1.63–33.02, p = 0.005; and OR = 10.29, 95% CI 1.45–72.81, p = 0.029).	The HLA-A*02:07, HLA-B*15:02, HLA-A*33:03, and HLA-B*15:02 alleles could be considered useful biomarkers for preventing cutaneous adverse drug reactions before LTG treatment in Thai patients. However, further studies of this type with larger numbers of patients are needed.
Significant HLA class I type associations with aromatic antiepileptic drug (AED)-induced SJS/TEN are different from those found for the same AED-induced DRESS in the Spanish population.	Ramírez et al. (2017)	Cases and controls	CBZ PHT LTG	HLA-A*02:01 HLA-A*11:01 HLA-B*38:01	A risk of association was observed between HLA-A*02:01/Cw15:02 and PHT-induced SJS/TEN (Group-B OR = 14.75, p = 0.009; G-C OR = 27.50, p < 0.001). HLA-B*38:01 and PHT-LTG (Group-A OR = 12.86, p = 0.012; G-B OR = 13.81, p = 0.002; G-C OR = 14.35, p < 0.001), and LTG (G-A OR = 147.00, p = 0.001; G-B OR = 115.00, p < 0.001; G-C OR = 124.70, p < 0.001). HLA-A*11:01 and CBZ (G-A OR = 63.89, p = 0.002; G-B OR = 36.33, p = 0.005; G-C OR = 28.29, p = 0.007).	HLA-A*02:01/Cw*15:02 alleles are associated with an increased risk of PHT-induced SJS/TEN, HLA-B*38:01 is associated with LTG- and PHT-induced SJS/TEN, and HLA-A*11:01 is associated with CBZ-induced SJN/TEN.
HLA-A*24:02 as a common risk factor for antiepileptic drug-induced cutaneous adverse reactions.	Shi et al. (2017)	Cases and controls	CBZ PHT LTG	HLA-A*24:02 HLA-B*15:02	Association was confirmed between HLA-B*15:02 with CBZ-induced SJS (p = 5.63 × 10^−15^), HLA-A*24:02 with CBZ-induced SJS (p = 0.015), LTG (p = 0.005) and PHT (p = 0.027). Positivity for HLA-A*24:02 and/or HLA-B*15:02 showed a sensitivity of 72.5% and a specificity of 69.0%.	HLA-A*24:02 is a genetic risk factor for aromatic antiseizure medications-induced SJS in the southern Han Chinese population and possibly in other ethnic populations. Pretreatment genetic testing is recommended in the southern Chinese population.
Pharmacogenomics predictors of aromatic antiepileptic drugs-induced SCARs in the Iraqi patients.	Ahmed et al. (2024)	Cases and controls	CBZ PHT LTG	*HLA-A∗24:02* *HLA-B∗15:02* *HLA-B∗40:02* *HLA-DRB1∗03:01*	HLA-A*24:02 and HLA-B*15:02 are associated with increased risk of ASMs-induced SJS (OR = 3.60, 95% CI 1.21–10.72 and OR = 4.41, 95% CI 1.18–16.47, respectively). *HLA-DRB1∗03:01* is associated with TEN (OR = 5.09; 95% CI 1.72–15.00). *HLA-B∗40:02* is associated with DRESS (OR = 29.33; 95% CI 3.50–245.32) induced by ASMs.	These alleles could be used as biomarkers for predicting and preventing ASMs-induced SCARs in genomic personalized medicine.

Abbreviations: ADRs, Adverse drug reactions; DRESS, drug rash with eosinophilia and systemic symptoms; SCARs, severe cutaneous adverse drug reactions; MPR, maculopapular rash; SJS/TEN, Stevens-Johnson syndrome (SJS) and toxic epidermal necrolysis (TEN); ASMs, antiseizure medications; CBZ, carbamazepine; PHT, phenytoin; LTG, lamotrigine; OR, odds ratio; 95% CI, 95% confidence interval.

## 6 Immunopathogenesis of hypersensitivity reactions

The mechanism of immunopathogenesis of SJS/TEN and DRESS/DIHS induced by ASMs is still unclear, so the most studied mechanisms are proposed. Aromatic ASMs and their metabolites behave as haptens that are phagocytosed by keratinocyte antigen-presenting cells (APCs) to degrade them into small fragments (antigens) of ASM. ASM antigens (ASM-Ag) would activate the HLA-B*15:02 allele located within one of the genes that code for the major histocompatibility complex (MHC) type I, then, MHC type I bound to ASM-Ag presents it on the cell surface of the keratinocyte and the cytotoxic T cell receptor (CD8^+^) recognizes it, generating a massive clonal expansion of these cells that accumulate in the damaged epidermis of the skin, and release perforin, granzyme B, granulysin (cytolytic protein) (Gibson et al., 2023), Fas ligand (FasL or CD95L), interferon gamma (IFN-γ), and tumor necrosis factor alpha (TNF-α) (Dodiuk-Gad et al., 2015; Sousa-Pinto et al., 2016; Su et al., 2017; Stewart et al., 2024). Additionally, monocytes and other cells produce IL-5 that activates CD8 T cells and Natural killer (NK) cells (Lee et al., 2024). TNF-α binds to the TNF receptor to activate procaspase 8, while perforin destroys the keratinocyte membrane, forming pores through which granzyme B enters, which also activates procaspase-8. Likewise, the Fas ligand of lymphocytes (FasL) binds to the Fas receptor associated with death domain-associated factors (FADD) that recruits procaspase-8, forming a signaling complex that promotes the activation of procaspase-8 in caspase-8 and this activates procaspase-3/7 in caspases-3/7. Additionally, caspase-8 cleaves the proapoptotic protein Bid (member of the Bcl-2 family) generating the truncated protein tBid that translocates to the outer membrane of the mitochondria, where they activate the BAX and BAK proteins, which undergo conformational changes to form pores in the outer mitochondrial membrane, releasing cytochrome c, forming the caspase-9-cytochrome c-Apaf-1 complex, from which caspase-9 is released, which stimulates caspases 3/7. Caspases 3/7 promote life-threatening keratinocyte apoptosis, necroptosis, and epidermal detachment (Ko et al., 2011; Kumar Das et al., 2014; Dodiuk-Gad et al., 2015; Estrella-Alonso et al., 2017; Charlton et al., 2020; Stewart et al., 2024). In addition, reactive oxygen species (ROS) formed within keratinocytes contribute to intracellular damage (Abe, 2008; Chung et al., 2008; Lee and Chung, 2013).

**FIGURE 2:**
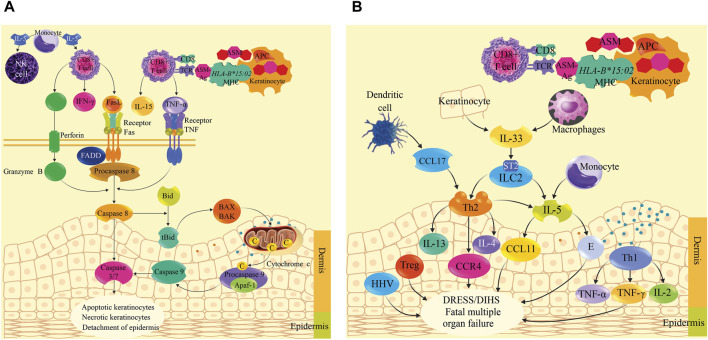
Immunopathogenesis mechanism of Stevens-Johnson syndrome (SJS)/toxic epidermal necrolysis (TEN) and drug reaction with eosinophilia and systemic symptoms (DRESS)/drug-induced hypersensitivity reaction (DIHS) induced by antiseizure medications. **(A)** shows the immunopathogenic process of SJS/TEN induced by aromatic ASMs. ASM is phagocytosed by keratinocyte antigen-presenting cells (APCs) to degrade it into ASM antigens (ASM-Ag). ASM-Ag activates HLA-B*15:02 located within one of the genes encoding the major histocompatibility complex (MHC) type I. MHC type I bound to ASM-Ag presents it on the cell surface of the keratinocyte and the cytotoxic T cell receptor (CD8^+^) recognizes it, then a massive clonal expansion of CD8^+^ is generated that accumulate in the damaged epidermis of the skin, and release perforin, granzyme B, granulysin (cytolytic protein), Fas ligand (FasL or CD95L), interferon gamma (IFN-γ), and tumor necrosis factor alpha (TNF-α). These cytokines activate caspases to cause apoptosis, keratinocyte necroptosis, and epidermal sloughing. Monocytes produce IL-5, which activates CD8 T cells and natural killer (NK) cells. **(B)** shows the immunopathogenic process of DRESS/DIHS. Keratinocytes and macrophages release IL-33, which binds to the ST2 receptor, activating type 2 innate lymphoid cells (ILC2). Plasma dendritic cells (DCs) produce chemokine ligand 17CC (CCL17) to recruit Th2 T-cells expressing chemokine receptor 4 (CCR4). Th2 cells and ILC2 produce IL-5 to induce eosinophil (E) activation and migration; in addition, Th2 cells release IL-4 and IL-13. Eosinophils produce CCL11 (eotaxin-1). IL-5 and CCL11 promote the local accumulation of harmful eosinophils. Th1 cells release TNFα, IFN-γ, IL-2, and IL-12. Human herpesvirus (HHV) reactivation and regulatory T cells (Tregs) are disrupted. All these cytokines and chemokines are responsible for DRESS/DIHS, which can lead to fatal multiorgan failure.

In DRESS/DIHS, CD4^+^ and CD8^+^ T cells, plasma dendritic cells (DC), regulatory T cells (Tregs), innate lymphoid cells type 2 (ILC2), and monocytes (M) accumulate in the dermis (Chen et al., 2023). Keratinocytes and macrophages release IL-33 and bind to the ST2 receptor to activate ILC2, meanwhile, DCs produce CC chemokine ligand 17 (CCL17) to recruit Th2 T-cells that primarily express chemokine receptor 4 (CCR4). Th2 cells and ILC2s produce IL-5 to induce eosinophil activation and migration (E), in addition, Th2 release IL-4 and IL-13. Eosinophils produce eotaxin-1 (known as CCL11), meanwhile, IL-5 and eotaxin-1 promote the local accumulation of harmful eosinophils. Additionally, Th1 cells release other cytokines such as TNFα, IFN-γ, IL-2 and IL-12, while human herpes virus (HHV) reactivation and alteration of regulatory T cells (Treg) occur. All these cytokines and chemokines are responsible for DRESS/DIHS induced by aromatic ASMs (Chen et al., 2023; Gibson et al., 2023) and can lead to fatal multi-organ failure (Böhm et al., 2018; Nogueiras, 2019). The immunopathogenesis of SJS/TEN is summarized in Figure 2A, and the proposal for DRESS is shown in Figure 2B.

## 7 Non-genetic factors associated with ADRs, and SCARs induced by aromatic antiseizure medications

The main non-genetic factors associated with adverse drug reaction and SCAR are age, concomitant diseases, polytherapy, high doses of ASMs, alcohol intake, sex and viral diseases (Bayane et al., 2024; Costa and Vale, 2024). In advanced age, there is a morphological change in hepatocytes and mitochondrial dysfunction (Schmucker, 2005), a decrease in the number of functional glomeruli due to nephrosclerosis (Denic et al., 2016), this decreases metabolism, extends the half-life, increases plasma levels of ASMs which leads to a risk of adverse drug reaction (Sánchez Romero et al., 2005). Comorbidities such as anxiety, depression, dementia, migraines, arthritis, heart disease, and peptic ulcers are up to eight times more common in people with epilepsy than in the general population (Keezer et al., 2016), which are strongly associated with a higher risk of ADRs (Giardina et al., 2018; Du et al., 2019).

Polytherapy among ASMs carries a higher risk of adverse drug reaction compared to monotherapy (Kumar et al., 2020; Kopciuch et al., 2022). Valproic acid, stiripentol, felbamate, and rufinamide are enzyme inhibitors that decrease metabolism and increase plasma levels of other ASMs (Benedetti, 2000; Alvarado et al., 2023b), for example, when the minimum toxic concentration of carbamazepine (12 mg/L) is exceeded, photosensitivity, eosinophilia and hepatotoxicity are observed (Kamitaki et al., 2021; Zgolli et al., 2024), meanwhile, phenytoin (20 mg/L) induces neurotoxicity (dizziness, nystagmus, ataxia and excessive sedation) (Dorado et al., 2013), gingival hyperplasia, hirsutism, and acne (Asadi-Pooya et al., 2021; Alvarado et al., 2023a; Zgolli et al., 2024). Additionally, it has been reported that patients with epilepsy are more susceptible to human immunodeficiency virus (HIV), cytomegalovirus or Epstein-Barr virus, which cause persistent brain infection, chronic neuroinflammation and seizures, this occurs in patients with weakened immune systems (Costa and Vale, 2024). These infections can inhibit liver enzymes, which affects the speed and extent of drug metabolism, generating supratherapeutic levels and increasing the risk of toxicity, which requires dose adjustment or selecting ASMs that are not metabolized by enzymes of the CYP-450 system (Galgani et al., 2018).

It is important to understand the interaction of non-genetic factors with genetic factors (polymorphisms in the *CYP2C9*, *CYP2C19*, and *HLA* genes) due to the possibility of increasing the risk of adverse drug reactions and SCARs, predicting them, and implementing preventive measures.

## 8 Clinical implications

This study has clinical implications by identifying risk alleles (predictive medicine) that allow for the prevention of hypersensitivity reactions (preventive medicine), and by personalizing and evaluating treatment discontinuation (genomic or precision personalized medicine).

Pharmacogenetic testing ordered by a neurologist can help identify patients with genetic alleles at higher risk for hypersensitivity reactions. This test should be performed before starting pharmacological treatment with antiseizure medications.

By identifying at-risk patients, neurologists can take preventive measures to minimize the severity of hypersensitivity reactions. Likewise, knowing the genotype and metabolic phenotype of patients will allow for personalizing or adjusting the dose from the start of drug treatment. At the same time, understanding the allelic variants CYP2C19*2, CYP2C9*3, and human leukocyte antigens (HLA) as pharmacogenomic biomarkers can be crucial in deciding whether to discontinue treatment, guide treatment with other antiseizure medications, or choose a safer therapeutic alternative for patients with epilepsy.

It is also necessary to indicate that this descriptive review has limitations that could lead to bias or confusion. The first limitation is the limited published scientific literature on allelic variants of pharmacogenes associated with Stevens-Johnson syndrome and toxic epidermal necrolysis induced by a group of antiseizure medications, with small patient samples and no statistical analysis of association. However, this descriptive study contributes to updating and synthesizing the knowledge on *CYP2C9*, *CYP2C19* and *CYP3A4* pharmacogenes associated with hypersensitivity reactions induced by aromatic antiseizure medications published to date. It will also be a scientific document to initiate studies in patients with epilepsy in Peru and Latin America.

## 9 Conclusions and future perspectives

Published scientific evidence demonstrates that CYP2C19*2, CYP2C9*3 and various HLA are associated with severe cutaneous adverse reactions, toxic epidermal necrolysis and Stevens-Johnson syndrome. Neurologists should consider these allelic variants as predictive and preventive genetic biomarkers of severe adverse reactions to carbamazepine, phenytoin, phenobarbital, and lamotrigine.

Furthermore, prospective multicenter and observational studies with larger numbers of patients are required to allow for the application of association statistics. This study is relevant for neurologists, who will have an academic tool to apply in their clinical practice. It will also constitute the first document for developing a Pharmacogenomic Guide that will allow the implementation of 4P medicine (predictive, preventive, personalized, and participatory) in health systems to improve the quality of life of patients with epilepsy, especially those in Peru and Latin America.
